# The Difference of Expression of 18 Genes in Axillary Invasion and Vascular Invasion Compared to Control Samples in Breast Cancer

**DOI:** 10.30699/ijp.2019.92094.1894

**Published:** 2019-08-01

**Authors:** Alireza Abdollahi, Sepideh Jahanian, Nima Hemmati, Hadis Mohammadpour

**Affiliations:** 1 Department of Pathology, School of Medicine, Tehran University of Medical Sciences, Tehran, Iran; 2 Student Research Committee, School of Medicine, Iran University of Medical Sciences, Tehran, Iran

**Keywords:** Breast cancer, Gene, Epithelial-mesenchymal transition, Lymphovascular invasion

## Abstract

**Background & Objective:**

Recent studies from gene profiling have revealed some genes that are overexpressed in the epithelial-mesenchymal transition (EMT) process and are responsible for its initiation and activation resulting in tumor progression and metastasis. The present study aimed to assess the role of genes involved in the EMT process and the association of these genes with axillary lymph node and vascular invasion in breast cancer (BC) patients.

**Methods:**

In this case-control study, the tumor samples were initially extracted from 33 BC patients. The samples of 15 BC tissues without vascular and axillary invasion were also prepared from the biobank as a control group. RNAs from both tumor and control samples were extracted and stabilized. For assessing overexpression in tumor tissues of selected 18 genes, the real time technique was employed.

**Results:**

There was a significant increase in MMP-2 gene fold expression in tumor cells with vascular invasion regardless of axillary involvement compared to the control group (*P*=0.0008) and also in the comparison of the control group with those with vascular invasion and not axillary lymph node involvement (*P*=0.003). In addition, gene fold expression of tissue inhibitors of metalloproteinase-1(TIMP-1) was decreased in axillary involving tumor cells compared to control group (*P*=0.045), and also in comparison with all samples that did not present any axillary lymph node involvements including the control group and the group with isolated vascular invasion (*P*=0.012).

**Conclusion:**

Overexpression of MMP-2 and under-expression of TIMP-1 were associated with more invasive behavior in breast tumor cells.

## Introduction

Breast cancer (BC) is a common malignant disease among both eastern and western women leading to high mortality rate, disability, and impaired quality of life (1). This malignant disorder has been identified as the second cause of cancer-related deaths followed by lung cancer in women accounting for 23% of cancer diagnoses (2). Despite a higher incidence of BC among developing countries, more than half of BC deaths are now thought to occur in the industrialized countries (3). The main etiology for BC mortality is due to the late diagnosis of metastases to distant sites especially to the vital organs; however, recently by applying screening tools, improvement of cancer-related healthcare, and development of adjuvant therapy, the mortality related to BC could be significantly controlled (4, 5). 

Lymphovascular invasion is a preceding step of metastasis and is considered when the neoplastic cells are seen in lymphatic and blood vessels. As indicated in previous studies, the lymphovascular invasion is related to a poorer survival (6), poorly differentiated tumors, distant metastasis, and recurrence (7), however, the particular molecular mechanism of lymphovascular invasion is not yet fully discovered (8, 9). 

The distant metastasis of the malignant tumors can be explained by various theories. In epithelial tumors such as breast tumor, the epithelial-mesenchymal transition (EMT) phenomenon can appropriately explain the processes of distant metastasis (10). Recent studies from gene profiling have revealed some genes that can be overexpressed within the EMT process causing its activation and leading to tumor progression and metastasis (11, 12). Moreover, the expression of these genes in the EMT process may be associated with other tumor-related behaviors including vascular and lymphatic invasions which play a critical role in planning distant metastasis (13). 

Previous studies showed an association of matrix metalloproteinase (MMP) and tissue inhibitors of metalloproteinase (TIMP) with metastasis in BC (14). Furthermore, some studies investigated the effect of blocking MMP-2 expression on cancer invasion (15). TIMP-1 seems to be a potential biomarker for diagnosis and prognosis of different malignancies (16).

The present study aimed to assess the profiling of the genes involved in EMT process and its association with axillary lymph node involvement and vascular invasion in BC patients. 

## Materials and Methods

**Patients and Sampling**

The tumor samples were extracted from 33 diagnosed BC patients with lymphovascular invasion kept at the Iran Tumor Biobank (Iran Cancer Institute). In addition, 15 control samples were randomly selected from BC tissues without vascular and axillary invasion, also provided from the tumor biobank. Informed consent was obtained from patients before the procedure.

The samples were kept in nitrogen after the surgery and were examined by a pathologist. Extracted RNAs from tumor and control samples were stabilized using the Ribo Ex preservation solution (Geneall, Daejeon, Korea). 

**RNA extraction**

To extract the RNAs from tissues, 1 mL Ribo Ex solution was transferred to the tube and then the tissue in the tube was homogenized and centrifuged for 10 seconds. Thereafter, 200 µL chloroform was added to the tube and the tube was centrifuged at 1700 g for 15 minutes. After adding chloroform, the superficial phase including RNAs was transferred into a new tube. Then, 500 mL isopropanol was poured into the new tube and centrifuged at maximal speed (14,000 rpm) for 20 min. There was a pellet barely visible at the base of each tube. The isopropanol was poured off and added with 1 ml of 75% ethanol in DEPC treated H_2_O and mixed gently. Then, the ethanol was poured off and let the pellets air-dry to store the RNAs. The quantity of RNAs was measured using NanoDrop 2000 Spectrophotometer. Also, the quality of the RNAs was tested by gel electrophoresis. The capacity cDNA reverse transcription kit (TaKaRa Bio Inc., Japan) was used for the quantitative conversion of total RNA to complementary DNA (cDNA). The products were amplified by polymerase chain reaction (PCR) and then were run on the gel. Gene expression was assessed by real time polymerase chain reaction (RT-PCR) using the specific primers. For analysis of the expression of the genes, the 2^-ΔΔCT ^method was used. Briefly, the mean target gene mRNA expression level for the mRNA measurements was calculated. The 2^-ΔΔCT ^method was used to calculate relative changes in gene expression determined from quantitative RT-PCR experiments. The data were presented as the fold change in target gene expression in tumors normalized to the internal control gene (*GAPDH*) and relative to the control group (matched normal as a calibrator). Results of the RT-PCR data were represented as C_T _values, where C_T _was defined as the threshold cycle number of PCRs at which amplified product was first detected. There was an inverse correlation between C_T _and amount of the target: lower amounts of the target correspond to a higher C_T _value, and higher amounts of the target have lower C_T _values. The average C_T _was calculated for both the target genes and *GAPDH*, and the ΔC_T _was determined as (the mean of the triplicate C_T _values for the target gene) minus (the mean of the triplicate C_T _values for *GAPDH*). The ΔΔC_T _represented the difference between the paired tissue samples, as calculated by the formula ΔΔC_T _= (ΔC_T _of tumor – ΔC_T _of normal). The N-fold differential expression in the target gene of a tumor sample compared to the control sample counterpart was expressed as 2^-ΔΔCT ^(16-18). 

Statistical Analysis

To describe quantitative variables, median and range were used and for qualitative variables frequency was used. To analyze the difference of variables a Mann-Whitney U test was performed. The data were entered and analyzed by SPSS version 17. 

Ethical Considerations

This study was approved by the ethics committee of the Tehran University of Medical Sciences. Informed consent was obtained from patients before the surgery.

## Results

A total of 48 samples was assessed and analyzed in this study. Among these samples, vascular invasion was seen in 68.75%, in which 43.75% had isolated vascular invasion and 25% had axillary lymph node invasion alongside the vascular invasion. Control samples were 31.25% of total samples. All of the samples with lymph node axillary invasion also had a vascular invasion. Patients’ age was lower than 35 years in 14%, 35 to 50 years in 40%, and higher than 50 years in 46%. Following the Kolmogorov-Smirnov test, the collected data regarding gene fold change were not normally distributed; therefore, we described the median and range for those data. Table (1) illustrates these indices regarding fold changes in different variables.

We used the Mann-Whitney U test for analyzing the data due to the non-normal distribution of our variables. The results of this analysis concerning the difference of gene fold expression in the control group and all patients with vascular invasion regardless of axillary involvement are presented in Table (2). MMP-2 gene fold expression was significantly higher in vascular invasion group (median=657.21) compared to control group (median=16.7) (*P*=0.008). 

The results of gene fold expression difference comparing the control group and group with isolated vascular invasion (without axillary invasion) are shown in Table (3). MMP-2 was significantly higher in isolated vascular invasion (median=981.63) compared to control group (median=16.7) (*P*=0.003). 

**Table1:** Gene fold indices assessed for each variable

	Gene	Median	Minimum	Maximum	Range
1	TIMP-1⌘	61.875	0.004	1096.59	1096.586
2	MMP-2★	256.498	0.33	54815	54814.67
3	Claudin	4.46	.061	13404.3	13404.239
4	MMP-9★	7.035	0.003	642.9	642.897
5	MEST1	0.432	0.003	14022.194	14022.191
6	Radixin	1.615	0.034	19.769	19.735
7	Cortactin	226.53	0.089	62038.073	62037.984
8	Cofilin	3.58	0.009	1802.5	1802.491
9	MEST2	0.355	0.001	45.36	45.359
10	EOMES⌂	11.198	0.2032	578.1	577.8968
11	MEST3	0.223	0.002	861.085	861.083
12	Villin	0.491	0.002	67.28	67.278
13	Gelsolin	7.8	0.05	11578.09	11578.04
14	FGF10	0.55	0.003	69.63	69.627
15	Fibronectin	469.81	0.098	41719.07	41718.972
16	Vimentin	88.44	0.096	1236.69	1236.594
17	AXL	31.81	0.25	13269.7	13269.45
18	GAS6	3.37	0.009	1802582	1802.573

★ MMP: Matrix metalloproteinase

⌘ TIMP: Tissue inhibitor of metalloproteinase

MEST: Mesoderm specific transcript

⌂ EOMES: Eomesodermin

FGF: Fibroblast growth factor

GAS: Growth arrest specific

**Table2:** Differences of gene fold expression in control group and vascular invasion group including isolated vascular invasion and concurrent vascular and axillary invasion

	Gene	Mean rank of control group N=15	Mean rank of vascular invasion group N=33	M-U*	P-value
1	TIMP-1⌘	27.27	23.24	206	0.356
2	MMP-2★	16.2	27.66	123	0.008
3	Claudin	20.32	24.89	179.5	0.288
4	MMP-9★	18.57	24.33	155	0.166
5	MEST1	26.21	21.55	172	0.270
6	Radixin	21.62	24.24	190	0.550
7	Cortactin	25.21	22.75	200	0.567
8	Cofilin	22.14	24.79	205	0.545
9	MEST2	23.20	23.65	228	0.916
10	EOMES⌂	21.57	23.65	197	0.624
11	MEST3	19.25	23.72	153	0.304
12	Villin	25.00	22.84	203	0.616
13	Gelsolin	21.64	2217	198	0.897
14	FGF10	22.43	23.97	209	0.720
15	Fibronectin	23.27	24.34	229	0.802
16	Vimentin	22.27	23.37	214	0.791
17	AXL	22.33	23.33	215	0.810
18	GAS6	20.21	25.61	178	0.218

* M-U: Mann-Whitney U Index

★ MMP: Matrix metalloproteinase

⌘ TIMP: Tissue inhibitor of metalloproteinase;

MEST: Mesoderm specific transcript;

⌂ EOMES: Eomesodermin;

FGF: Fibroblast growth factor;

GAS: Growth arrest specific

**Table3:** Differences of gene fold expression in control group and vascular invasion group including only isolated vascular invasion cases

	Gene	Mean rank of control group N=15	Mean rank of isolated vascular invasion group N=21	M-U*	P-Value
1	TIMP-1⌘	18.53	18.48	157	0.987
2	MMP-2★	12.07	22.45	61	0.003
3	Claudin	14.54	19.58	98.5	0.146
4	MMP-9★	13.50	19.58	84	0.074
5	MEST1	19.36	17.10	128	0.522
6	Radixin	15.46	18.76	110	0.348
7	Cortactin	18.36	17.76	142	0.866
8	Cofilin	16.07	1929	120	0.363
9	MEST2	18.37	17.73	144.5	0.855
10	EOMES⌂	15.50	18.90	112	0.327
11	MEST3	13.96	18.03	89.5	0.235
12	Villin	18.50	16.80	126	0.624
13	Gelsolin	16.79	18.00	130	0.726
14	FGF10	17.29	18.48	137	0.736
15	Fibronectin	17.40	19.29	141	0.596
16	Vimentin	15.87	18.79	118	0.395
17	AXL	16.60	18.21	129	0.640
18	GAS6	14.86	20.10	103	0.138

* M-U: Mann-Whitney U Index

★ MMP: Matrix metalloproteinase

⌘ TIMP: Tissue inhibitor of metalloproteinase

MEST: Mesoderm specific transcript

⌂ EOMES: Eomesodermin

FGF: Fibroblast growth factor

GAS: Growth arrest specific

Table (4) demonstrates the difference of gene fold expression between the group with axillary lymph node invasion and groups without axillary lymph node invasion (including controls and those with exclusive vascular invasion). TIMP-1 was significantly lower in axillary invasion group (median=7.78) compared to controls and vascular invasion groups combined (median=90.44) (*P*=0.012).

The difference of the gene fold expression in the group with axillary invasion and control group is presented in Table (5). TIMP-1 was significantly lower in axillary invasion group (median=7.78) compared to controls (median=16.7) (*P*=0.045).

**Table 4:** Gene fold expression differences in groups without axillary lymph node invasion and groups with axillary lymph node invasion

	Gene	Mean rank of control group and isolated vascular invasion group N=36	Mean rank of group with axillary lymph node invasion N=12	M-U*	P-Value
1	TIMP-1⌘	27.42	15.75	111	0.012
2	MMP-2★	24.34	23.00	198	0.770
3	Claudin	24.47	20.75	171	0.409
4	MMP-9★	23.33	20.00	154	0.456
5	MEST1	24.54	17.60	121	0.140
6	Radixin	24.06	21.92	185	0.635
7	Cortactin	24.80	19.36	147	0.241
8	Cofilin	25.49	19.67	158	0.205
9	MEST2	22.59	26.41	160.5	0.410
10	EOMES⌂	23.97	20.00	154	0.383
11	MEST3	22.19	23.33	182	0.792
12	Villin	23.99	22.13	187.5	0.680
13	Gelsolin	22.65	19.56	131	0.511
14	FGF10	23.04	24.95	176.5	0.680
15	Fibronectin	24.72	21.64	172	0.514
16	Vimentin	24.41	18.64	139	0.205
17	AXL	23.59	21.18	167	0.597
18	GAS6	25.31	20.17	164	0.262

* M-U: Mann-Whitney U Index

★ MMP: Matrix metalloproteinase

⌘ TIMP: Tissue inhibitor of metalloproteinase

MEST: Mesoderm specific transcript

⌂ EOMES: Eomesodermin;

FGF: Fibroblast growth factor

GAS: Growth arrest specific

**Table 5:** Gene fold changes differences in control group and axillary invasion group

	Gene	Mean rank of control group N=15	Mean rank of axillary invasion group N=12	M-U*	P-Value
1	TIMP-1⌘	16.73	10.58	49	0.045
2	MMP-2★	12.13	16.33	62	0.172
3	Claudin	13.29	13.75	81	0.877
4	MMP-9★	12.57	13.55	71	0.743
5	MEST1	14.36	9.90	44	0.128
6	Radixin	13.15	12.83	76	0.913
7	Cortactin	14.36	11.27	58	0.298
8	Cofilin	13.57	13.42	83	0.959
9	MEST2	12.83	14.41	75.5	0.604
10	EOMES⌂	13.57	12.27	69	0.661
11	MEST3	11.79	13.21	63.5	0.624
12	Villin	14.00	12.92	77	0.719
13	Gelsolin	12.36	11.44	58	0.753
14	FGF10	12.64	13.45	72	0.784
15	Fibronectin	13.87	13.00	77	0.775
16	Vimentin	14.40	12.27	69	0.484
17	AXL	13.73	13.18	79	0.856
18	GAS6	12.86	14.25	75	0.643

* M-U: Mann-Whitney U Index

★ MMP: Matrix metalloproteinase;

⌘ TIMP: Tissue inhibitor of metalloproteinase

**MEST: Mesoderm specific transcript;**

⌂ **EOMES: Eomesodermin;**

**FGF: Fibroblast growth factor;**

**GAS: Growth arrest specific**

## Discussion

According to the present study, there was a significant increase in MMP-2 gene fold expression in tumor cells with vascular invasion compared to control samples. This was significant comparing the control group with all the samples with vascular invasion regardless of axillary lymph node involvement (*P*=0.0008) and vascular invasion without axillary lymph node involvement (*P*=0.003).

Furthermore, it was observed that in tumor cells with axillary lymph node invasion, tissue inhibitors of metalloproteinase-1 (TIMP-1) gene fold expression was lower compared to the control group (*P*=0.045).

These tumor cells also demonstrated lower increase in TIMP-1 gene fold expression levels when compared to the groups without axillary lymph node involvement including the control group and the group with isolated vascular invasion (*P*=0.012).

The MMPs are known to have a role in extracellular matrix degeneration (17) and so can contribute to the mechanism on tumor invasion. They are found in both normal cells and neoplastic cells (18) and therefore play a role in some other cell functions such as wound healing, angiogenesis, apoptosis, and immune response as well as tumor invasion (17).

On the other hand, TIMPs are proteins that can bind to MMPs and inhibit their action (19). TIMP-1, which was assessed in the present study, is known to inhibit MMP-9 whereas MMP-2 is inhibited by TIMP-2 (20). So far, various studies have demonstrated the role of MMPs and their inhibitors including TIMPs in cancer progression and metastasis. 

According to a study by Pellikainen et al. in 2004 (17), the expression levels of MMP-2 and MMP-9 were both increased in the cells of breast tumors. However, we did not find a significant increase in MMP09 gene fold expression between groups with invasion and control group in the present study. Although the difference of MMP-9 expression was not significant in our study, the results of previous studies regarding MMP-2 are in accordance with those of the current study. This could be justified by the difference of methods used to measure the expressions of genes, since in this study the researchers used RT-PCR method and quantitative measurements of gene expressions but in the study by Pellikainen et al., the differences of expressions were measured by categorical analysis with a cut-off point of 80% for MMP-2 and 85% for MMP-9 of cytoplasmic expressions in immunohistochemistry test. Although the expression of MMP-9 increased in the vascular invasion in this study, the difference was not significant. 

The study by Figueira et al. (2009) showed higher amounts of MMP-2, MMP-9, TIMP-1, and TIMP-2, and reversion inducing cysteine-rich protein (RECK) levels in more aggressive cells compared to the less aggressive cancer cells (21). In our study, the amounts of TIMP-1 were significantly lower in patients with axillary lymph node invasion compared to control samples. But as it was noted before, the amounts of MMP-2 increased in samples with vascular invasion and groups with axillary and vascular invasion compared to the control group. This might be explained by the fact that the grouping method was different in the study by Figueira et al. and the current research; while they based their grouping on the cell line of malignant cells and the histopathology, we based it on axillary invasion, vascular invasion, and controls. Although both studies showed that all of the mentioned genes increase proportional to normal expressions, it seems that the findings of the study by Figueira et al. regarding TIMP-1 gene cannot be completely true due to the lack of controls in their study.

Owing to the fact that the present study aimed to assess the genes that might be involved in the invasive behavior of BC cells through utilizing the EMT process, some of the studies that have discovered the association between these genes and lymph node invasion in cancers other than breast are discussed below; these findings support the key role of MMP and TIMP genes in the process of metastasis.

In a study conducted by Yao et al. (2017), high amounts of MMP-2 and MMP-9 in early gastric cancer cells were significantly associated with lymph node metastasis and ulceration. The mentioned study also showed that the prognostic value of MMP-2/MMP-9 overexpression was independent of other tumor prognostic factors like tumor size, depth, and the number of lymph nodes involved in lymph node metastasis (22). Another study by Wang et al. (2011) suggested that upregulation of platelet-derived growth factor-D enhances the expression of MMP-2 and MMP-9 contributing to the invasion of ovarian tumor cells (23). Huang et al. (2013) also found a significant difference of MMP-2 expressions in lymph node metastatic cells of the patients with nasopharyngeal cancer metastasized to cervical lymph nodes (24). Among head and neck cancers, a study by Zhou et al. (2010) showed an association between MMP-2 and MMP-9 overexpression in cervical lymph node metastasized tongue cancers (25). 

Considering the BCs, the existing evidence supports the overexpression of MMP-2 gene and invasive behavior (26-28), which is in line with the findings of our study. 

Despite the known role of TIMP-1 as being MMP inhibitors, it seems that the function of this protein is more complicated as some studies have shown that high TIMP levels tend toward cancer progression.

A study by D’Angelo et al. (2014) found a significant association between TIMP-1 overexpression and EMT-like conversions such as phenotypic changes of vascular cells leading to adhesion molecule loss (29), which can explain the beginning of invasion as described in the EMT process theories. 

The study of Clarke and et al. (2015) showed that the inhibitions of TIMP-1 and -2 are responsible for the invasion of BC cells, probably due to their inhibitory effects on MMPs (16). Meanwhile, our study revealed that the relative decrease of TIMP-1 in comparison to control group was associated with the invasive behavior of the tumor. In a study by Bernert et al. (2011), the induction of TIMP-1 resulted in the invasive capability of BC cells by suppressing the hyaluronan synthase 2 (30). 

In another study related to colorectal cancer by Song et al. (2016), the human cancer cells showed a significant increase in TIMP-1 levels compared to controls. In addition, the lymph node-positive patients showed a significantly higher amount of TIMP-1, which proves its role in invasion and metastasis (31). 

In contrast to many studies showing high TIMP-2 expression in neoplastic cells with lymph node invasions, a study by Iniesta P. et al. (2007) confirmed a decrease in TIMP-2 measurements along with increased MMP-2 levels concurrently in non-small-cell lung carcinoma (NSCLC) patients (32). Interestingly, TIMP-2, regardless of its name, increased MMP-2 levels in animal experiments (29). 

The association of TIMPs with the progression of cancer seems to be due to other functions of these molecules. TIMP-1 promotes angiogenesis by upregulating vascular endothelial growth factor and inhibits apoptosis. TIMP-2 also inhibits apoptosis and promotes neoplastic growth (33-35). It has been suggested that the integrity of the cellular matrix is preserved when the MMP/TIMP levels are balanced (36). 

A study by Valente et al. (1998) on melanoma cells confirmed the overexpression of TIMP-2 cells, which was also associated with less migration and endothelial invasion compared to control cells (37). 

In a study by Batra et al. (2012), cancer cell invasion and tumor gelatinase (MMPs) activity was inhibited by PEGylating (conjugating with polyethylene glycol) recombinant human TIMP-1 (38). This could be an emphasis on the inhibitory role of TIMPs. 

Ikenaka et al. (2003) observed that the amounts of MMP-2 and MMP-9 decreased in the tumor cells of transgenic mice following ectopic TIMP-1 due to its inhibitory effect on cell growth and angiogenesis (39). 

As mentioned before and in contrast to most studies, the present study found lower levels of TIMP-1 in samples with axillary invasion compared to control group and vascular invasion group without axillary lymph node involvement. This finding could be explained by the findings of various studies regarding TIMPs. For example, a study of pancreatic tumor cells by Rigg et al. (2001) showed that although TIMP-1 levels are overexpressed in malignant cells, it appears that these proteins change the tumor invasive behavior as it reduces tumor cells reaching out to distant organs (40).

The sample size of this study was limited and data were not normally distributed. Therefore, we suggest designing a study with larger sample size and normal distribution. Unfortunately, the authors cannot calculate the minimum sample size required for future studies but we recommend other researchers to choose complete datasets. It should also be noted that the researchers did not consider distant metastasis in the present study. So, a complementary study to investigate this variable can be helpful.

## Conclusion

In conclusion, the present study demonstrated that cells with more invasive behavior express higher MMP-2 and lower TIMP-1 levels. However, the prognostic value of these molecules is not fully understood. As the role of these molecules in the process of invasion and tumor progression becomes clearer, more direct studies can be conducted to use them as target therapies or markers in the future.
